# Dietary Collagen Hydrolysates Retard Estrogen Deficiency-Induced Bone Loss through Blocking Osteoclastic Activation and Enhancing Osteoblastic Matrix Mineralization

**DOI:** 10.3390/biomedicines10061382

**Published:** 2022-06-10

**Authors:** Soo-Il Kim, Sin-Hye Park, Woojin Na, Yong Chul Shin, Moon-Sik Oh, Young Eun Sim, Yulong Zheng, Ae Hyang Kim, Il-Jun Kang, Young-Hee Kang

**Affiliations:** 1 Department of Food Science and Nutrition, Korean Institute of Nutrition, Hallym University, Chuncheon 24252, Korea; ky4850@naver.com (S.-I.K.); shpark88@hallym.ac.kr (S.-H.P.); nsm0729@hanmail.net (W.N.); 2569227@naver.com (M.-S.O.); sim2015@hallym.ac.kr (Y.E.S.); zyl1994@naver.com (Y.Z.); ijkang@hallym.ac.kr (I.-J.K.); 2 Amicogen Inc., Healthcare & Nutrition Lab., Jinju 52840, Korea; ycshin@amicogen.com (Y.C.S.); ahkim@amicogen.com (A.H.K.)

**Keywords:** Pangasius hypophthalmus fish skin collagen hydrolysate, ovariectomy, postmenopausal osteoporosis, glycine–proline–hydroxyproline tripeptide, collagen, osteoblasts

## Abstract

Osteoporosis manifest in postmenopausal women is an osteolytic disease characterized by bone loss, leading to increased susceptibility to bone fractures and frailty. The use of complementary therapies to alleviate postmenopausal osteoporosis is fairly widespread among women. The current study examined that Pangasius hypophthalmus fish skin collagen hydrolysates (fsCH) inhibited ovariectomy (OVX)-induced bone loss by conducting inter-comparative experiments for anti-osteoporotic activity among 206–618 mg/kg fsCH, 2 mg/kg isoflavone, 15 mg/kg glycine–proline–hydroxyproline (GPH) tripeptide, and calcium lactate. Surgical estrogen loss of mice for 8 weeks reduced serum 17β-estradiol levels with uterus atrophy, which was ameliorated by orally administering fsCH or isoflavone to mice. Similar to isoflavone, fsCH containing GPH-enhanced bone mineral density reduced levels of cathepsin K and proton-handling proteins, and elevated collagen 1 level in OVX bones. The treatment with fsCH and isoflavone enhanced the serum levels of collagen synthesis-related procollagen type 1 carboxy/amino-terminal propeptides reduced by OVX, whereas serum levels of osteocalcin and alkaline phosphatase, as well as collagen breakdown-related carboxy/amino-terminal telopeptides of type 1 collagen were reduced in OVX mice treated with fsCH, isoflavone, and calcium lactate. The trabecular bones were newly formed in OVX bones treated with isoflavone and fsCH, but not with calcium lactate. However, a low-dose combination of fsCH and calcium lactate had a beneficial synergy effect on postmenopausal osteoporosis. Furthermore, similar to isoflavone, 15–70 μg/mL fsCH, with its constituents of GPH and dipeptides of glycine–proline and proline–hydroxyproline, enhanced osteogenesis through stimulating differentiation, matrix mineralization, and calcium deposition of MC3T3-E1 osteoblasts. Accordingly, the presence of fsCH may encumber estrogen deficiency-induced bone loss through enhancing osteoclastogenic differentiation and matrix collagen synthesis. Therefore, fsCH may be a natural compound retarding postmenopausal osteoporosis and pathological osteoresorptive disorders.

## 1. Introduction

Primary osteoporosis is defined as a microarchitectural deterioration of bone tissues that leads to increased bone fragility and fractures [1]. Vertebral fractures represent the most frequent complication associated with osteoporotic diseases [2]. Postmenopausal osteoporosis, type 1 primary osteoporosis, occurs typically in postmenopausal women [3,4]. Senile osteoporosis, primary type 2 osteoporosis, is manifest in both females and males aged 70 years or over [5]. The risk of osteoporotic diseases can be lowered by taking medications including calcium, vitamin D, bisphosphonates, and denosumab [6,7,8]. A higher intake of carotenoids of β-carotene and β-cryptoxanthin is known to be associated with decreased osteoporotic fracture risk [9,10]. Daily administration of β-cryptoxanthin inhibits osteoclastic activation and the reduction of bone volume in estrogen-deficient mice [9]. The reduction of the estrogen level at menopause is one of the strongest risk factors for developing postmenopausal osteoporosis [11]. In postmenopausal osteoporosis, estrogen deficiency results in high bone turnover and low bone mass [11,12]. Accordingly, estrogen replacement therapy is one of the most effective treatments for the prevention of adverse postmenopausal alterations [13,14]. However, hormone replacement therapy contributes to the increased risk of breast cancer and malignancy, which hampers its wide usage [15,16]. Potential pharmacological therapies for postmenopausal osteoporosis include agents that improve bone strength, thereby leading to more efficient recovery of bone mass [17,18].

Concerns about the increased risk of long-term hormone therapy have prompted an increase in the use of harmless and affordable alternative therapies [19]. Naturally occurring nonsteroidal compounds have estrogen-like biological activity, primarily through binding to estrogen receptors [20,21]. Isoflavones are a type of naturally occurring isoflavonoid, many of which act as phytoestrogens in mammals [22]. Formononetin, an isoflavone, displays beneficial effects on the mechanical properties and chemical composition of bones in ovariectomized (OVX) rats [23]. Numerous studies have focused on revealing the inhibition of OVX-induced osteoclastogenesis by plant compounds. The apple polyphenol phloretin inhibits estrogen deficiency-induced osteoclastogenic osteoporosis in mice [24]. In addition, the green tea polyphenol epigallocatechin-3-gallate (EGCG) suppresses OVX-induced bone loss by inhibiting resorption and improving bone microarchitecture [25,26]. Furthermore, oral administration of plant extracts prevents OVX-induced bone loss by blocking osteoclast differentiation and activation [27,28]. Accordingly, potential plant agents targeting osteoclastic activation may display favorable effects in combating estrogen deficiency-induced resorptive bone diseases. However, their action mechanisms for manipulating bone-specific remodeling process are undefined under the OVX condition. There are multiple targets and specific pathways within osteoclastic cells involved in OVX-triggered resorptive bone loss [29,30].

The use of complementary therapies to alleviate postmenopausal osteoporosis is fairly widespread among women. Several studies have reported a potential utility of collagen hydrolysates in the treatment of osteoarthritis and osteoporosis [31,32]. On the contrary, the consumption of collagen hydrolysates exerts no significant effects on bone metabolism in postmenopausal women with low mineral density [33]. Based on the opposite effects of collagen hydrolysates on bone health, the current study evaluated that novel Pangasius hypophthalmus fish skin collagen hydrolysates (fsCH) protected postmenopausal bone loss. This study conducted inter-comparative experiments for anti-osteoporotic activity among fsCH, isoflavone, glycine–proline–hydroxyproline (GPH) tripeptide, and calcium lactate. In our previous study [34], the crude proteins formed ~97% in fsCH, and the contents of collagen tripeptides and GPH were 32.67 ± 0.22% and 3.22 ± 0.02%, respectively. GPH is considered to be a collagen-specific sequence, and is initially hydrolyzed to the dipeptide proline–hydroxyproline (PH) by the epithelium-bound aminopeptidase on the intestinal brush border membrane [35,36]. PH, a major active constituent of collagen-derived peptides, can be transported into enterocytes via the H+-coupled oligopeptide transporter [35]. Further, this study examined whether fsCH enhanced osteogenesis through stimulating differentiation and mineralization of mature osteoblasts. The osteoblastogenesis of fsCH was inter-compared with that of isoflavone, GPH, and dipeptides of PH and glycine–proline (GP). This study found that, similar to isoflavone, fsCH containing GPH might enhance bone formation reduced by OVX through improving collagen synthesis, osteoblastic activation, and matrix mineralization. The osteogenic activity of calcium lactate in postmenopausal osteoporosis was synergically complemented by fsCH.

## 2. Materials and Method

### 2.1. Materials

Dulbecco’s modified Eagle’s medium (DMEM), minimum essential medium Alpha modification (α-MEM), and calcium lactate were purchased from Sigma-Aldrich Chemical (St. Louis, MO, USA), as were all other reagents, unless specifically stated elsewhere. Fetal bovine serum (FBS), penicillin–streptomycin, and trypsin-EDTA were purchased from Lonza (Walkersville, MD, USA). Antibodies of mouse carbonic anhydrase II (CAII) and mouse bone morphogenetic protein 2 (BMP-2) were provided by Abcam Biochemicals (Cambridge, UK). Antibodies of mouse vacuolar-type H(+)- ATPase (V-ATPase), mouse cathepsin K, mouse collagen type I, mouse bone sialoprotein (BSP) II, and mouse osteopontin (BSP I) were obtained from Santa Cruz Biotechnology (Santa Cruz, CA, USA). Horseradish peroxidase (HRP)-conjugated goat anti-rabbit IgG, donkey anti-goat IgG, and goat anti-mouse IgG were supplied from Jackson ImmunoResearch Laboratory (West Grove, PA, USA). GPH standard was obtained from Bachem (Bubendorf, Switzerland), and isoflavone powder was obtained from Seorim Bio Inc. (Chuncheon, Korea). The collagen hydrolysates fsCH were derived from fish skin gelatin, and digested with non-pathogenic Bacillus collagenase-type protease (Amicogen Inc., Jinju, Korea). The preparation and HPLC analysis of fsCH were as previously detailed [34].

### 2.2. Animals and Ovariectomy (OVX)

To investigate the inhibitory effects of fsCH on osteoporotic activity in an estrogen-deficient animal model, this study introduced experimental ovariectomy-mimicking estrogen deprivation or senescent menopause. C57BL/6 mice (11 weeks of age, 20–25 g) were obtained from DBL (Eumsung, Korea), and kept on a 12-h light/dark cycle at 20–25 °C with 60% relative humidity under specific pathogen-free conditions. Mice were fed a non-purified diet (RodFeed, DBL) during the 8-week experimental period, with free access to water ad libitum at the animal facility of Hallym University. The animals were allowed to acclimatize for a week before beginning the experiments. All animal experiments were performed in accordance with the University’s Guidelines for the Care and Use of Laboratory Animals, approved by the Committee on Animal Experimentation of Hallym University (permission number: Hallym2020-15).

For the OVX surgery, 11-week-old female animals were anesthetized using a ketamine/Rompun cocktail (40 mg ketamine/kg and 10 mg rompun/kg body weight) for either a sham operation (Sham) or bilateral oophorectomy (OVX). Mice receiving surgical OVX were orally treated with 2 mg/kg isoflavone, 15 mg/kg GPH, 206 mg/kg calcium lactate, and 206–618 mg/kg fsCH once a day for 8 weeks (9–10 mice of each group). After 8 weeks of treatment, blood samples and uterus tissues were collected, and serum samples obtained by centrifugation (3000 rpm, 10 min) were stored at −70 °C prior to analyses.

### 2.3. Biochemical Assessment of Bone Minerals and Plasma Lipids

Serum levels of total cholesterol (TC), triglyceride (TG), high-density lipoprotein cholesterol (HDL-C), and low-density lipoprotein cholesterol (LDL-C) were analyzed by using an Automated Clinical Chemistry Analyzer (DRI-CHEM NX500, Fuji Film, Tokyo, Japan), according to the manufacturer’s instructions. Table 1 shows the lipid profile data with diverse dietary interventions after OVX surgery. There was significant increase in serum levels of total cholesterol, triglyceride, and LDL-cholesterol observed at 8 weeks after OVX, whereas HDL-cholesterol level declined (Table 1). The overall lipid profiles were not improved in OVX mice treated with fish collagen peptides, including fsCH, with the exception of isoflavone (Table 1).

The bone mineral density (BMD) and bone mineral content (BMC) of mouse femoral and tibial bones were determined with a PIXImus mouse densitometer (GE Lunar, Waukesha, WI, USA). BMD calculated by dividing BMC (mg) by the projected bone area (cm^2^) was assessed in the femoral and tibial regions.

### 2.4. Histological Examination of Uterus and Femoral Bone

Uterus and femoral bone tissues were obtained from three mice in each group. After being washed with saline, the uterine tissues were fixed in 10% neutral buffered formalin for 24 h. Femoral bone tissues were decalcified in decalcifying solution (Sigma-Aldrich Chemicals, St. Louis, MO, USA), and dehydrated in a graded series of ethanol solutions for 18 h. Femoral bone tissues were then embedded in paraffin and cut into 7 μm sections in thickness. The sections were placed on glass slides, deparaffinated, and hydrated with xylene and graded alcohol. Tissues were stained using a modified Harris hematoxylin and Shandon instant eosin (H&E) for microscopic observation. After each slide was mounted in VectaMount mounting medium (Vector Laboratories, Burlingame, CA, USA), images were taken using an Axiomager optical microscope system (Zeiss, Oberkochen, Germany).

### 2.5. Enzyme-Linked Immunosorbent Assay (ELISA)

Serum levels of 17*β*-estradiol and osteoprotegerin (OPG), and receptor activator of nuclear factor kappa-Β ligand (RANKL) were determined by using ELISA kits (R&D Systems, Minneapolis, MN, USA), according to the manufacturer’s instructions. In addition, serum levels of procollagen type 1 carboxy-terminal propeptide (PICP), procollagen type 1 amino-terminal propeptide (PINP), carboxy-terminal telopeptide of type 1 collagen (CTX-1), and amino-terminal telopeptide of type 1 collagen (NTX-1) were measured by using ELISA kits (Novus Biologicals, Centennial, CO, USA), according to the manufacturer’s instruction. Serum and bone levels of osteocalcin were examined by using ELISA kits (Life Technologies, Carlsbad, CA, USA).

### 2.6. Histological Tartrate-Resistant Acid Phosphatase (TRAP) Staining of Femoral Bone

For the histological TRAP staining, femoral bone tissues were decalcified, dehydrated, and then embedded in paraffin, and cross-sectionally cut into sections measuring 5 μm in thickness. The TRAP staining was conducted by using a leukocyte acid phosphatase kit (Sigma-Aldrich Chemicals, St. Louis, MO, USA), according to the manufacturer’s instructions. The femoral tissue samples were incubated for 20 min in 50 mM sodium acetate and 40 mM potassium sodium tartrate buffer (pH 5.0), and further incubated for 15 min in the same buffer containing 2.5 mg/mL Naphthol AS-BI phosphate and 0.5 mg/mL Fast Garnet GBC. After each slide with tissue sections was mounted in VectaMount mounting medium, images were taken using an Axiomager optical microscope system.

### 2.7. MC3T3-E1 Cell Culture and Osteoblastic Differentiation

The MC3T3-E1 cell line (ATCC CRL-2593) was obtained from the American Type Culture Collection (Manassas, VA, USA), and cultured in α-MEM supplemented with 10% FBS, 100 U/mL penicillin, and 100 μg/mL streptomycin at 37 °C with 5% CO_2_ in air. To differentiate MC3T3-E1 cells into osteoblasts, cells were seeded on a 24-well plates at a density of 6.5 × 10^4^ cells, and cultured in α-MEM (differentiation media) supplemented with 10 mM β-glycerol phosphate, 50 μg/mL ascorbic acid, and 100 nM dexamethasone for up to 21 days in the presence of 10 μg/mL isoflavone, 2.5 μg/mL GPH, 2.5 μg/mL proline–hydroxyproline (PH), 2.5 μg/mL glycine–proline (GP), and 15–70 μg/mL fsCH. The media for cells were freshly replaced every three days.

The cytotoxicity of isoflavone, GPH, PH, GP, and fsCH was assessed by MTT assay, based on the mitochondrial-dependent reduction of MTT to formazan crystal. MC3T3-E1 cells were seeded in 24-well plate at density 6.5 × 10^4^ cells, and cultured in differentiation media for 3 or 21 days in the absence and presence of 10 μg/mL isoflavone, 2.5 μg/mL GPH, 2.5 μg/mL PH, 2.5 μg/mL GP), and 15–70 μg/mL fsCH. After cell culture with isoflavone, GPH, PH, GP, and fsCH, 1 mg/mL MTT reagent was added to cells, which were then incubated for 3 h at 37 °C with 5% CO_2_. Isopropanol was added to shed the formation of an insoluble purple formazan product. Optical density was measured by using a microplate reader at λ = 570 nm, corrected by reference wavelength at 690 nm.

### 2.8. Measurement of Activity of Serum and Osteoblast Alkaline Phosphatase (ALP)

The ALP activity was performed in serum samples. The ALP activity of MC3T3-E1 cells was performed on day 7 during differentiation. Osteoblastic cells were lysed in 1.0% Triton X-100. For the measurement of ALP levels, serum and osteoblast lysates were incubated with 0.5 M Tris-HCl buffer (pH 9.9) containing 6 mM *p*-nitrophenyl phosphate and 1 mM MgCl_2_ at 37 °C for 2 h. The absorbance was read at λ = 405 nm in a microplate reader.

### 2.9. Western Blot Analysis

Equal amounts of crude tissue extract proteins and MC3T3-E1 cell lysate proteins were electrophoresed on 6–12% SDS-PAGE gels, and transferred onto a nitrocellulose membrane. Non-specific binding was blocked by soaking membranes in a TBS-T buffer [50 mM Tris-HCl (pH 7.5), 150 mM NaCl and 0.1% Tween 20] containing 3% bovine serum albumin or 5% non-fat milk for 3 h. The membranes were incubated with anti-mouse CAII, V-ATPase, cathepsin K, collagen type 1, BMP-2, BSPII, and osteopontin as a primary antibody. The membranes were then incubated with goat anti-rabbit IgG conjugated to HRP as a secondary antibody. The protein levels on gels were determined by using Supersignal West Pico Chemiluminescence detection reagents (Pierce Biotechnology, Rockford, IL, USA) and Konica X-ray film (Konica, Tokyo, Japan). Incubation with an antibody of mouse β-actin was conducted as a comparative control.

### 2.10. Alizarin Red S Staining

For the measurement of calcium deposits, MC3T3-E1 cells were seeded on a 24-well plate at a density of 6.5 × 10^4^ cells in differentiation media for 21 days with and without 10 μg/mL isoflavone, 2.5 μg/mL GPH, 2.5 μg/mL PH, 2.5 μg/mL GP, and 15–70 μg/mL fsCH. The medium culture was freshly changed every 3 days, and Alizarin red S staining was conducted on day 21. Cells were rinsed in cold PBS, fixed with 4% formaldehyde at room temperature for 15 min, and stained with 40 mM Alizarin red S dye (pH 4.2) for 10 min. Calcium deposits were observed under light microscopy.

### 2.11. Statistical Analyses

The results were expressed as means ± SEM for each treatment group in each experiment. Statistical analyses were performed using the Statistical Analysis Systems statistical software package (SAS Institute, Cary, NC, USA). Significance was determined by one-way analysis of variance, followed by Duncan’s range test for multiple comparisons. Differences were considered significant at *p* < 0.05.

## 3. Results

### 3.1. Recovery of Uterine Size and Serum 17β-Estradiol Level by fsCH

**Figure 1:**
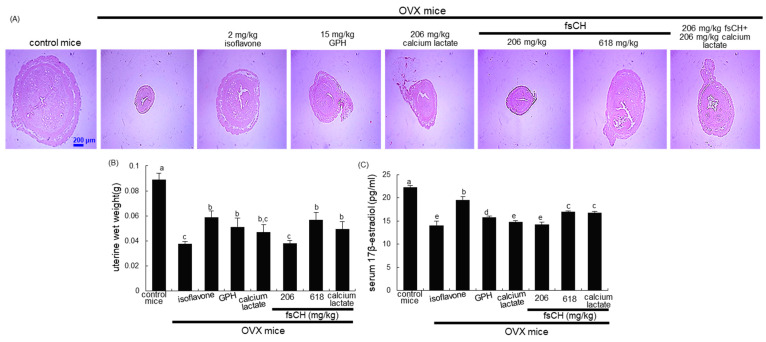
Uterus transverse section (**A**), wet weight of uterine tissues (**B**), and serum 17β-estradiol level (**C**) of ovariectomized (OVX) mice treated with various compounds daily for 8 weeks. OVX C57BL/6 female mice were orally administrated with 2 mg/kg isoflavone, 15 mg/kg glycine–proline–hydroxyproline tripeptide (GPH), 206 mg/kg calcium lactate, and 206–618 mg/kg Pangasius hypophthalmus fish skin hydrolysates (fsCH) daily for 8 weeks. Cross-sectional images of the uterine horn were obtained by staining with H&E, and visualized under light microscopy (**A**). Scale bar = 200 μm. Serum 17β-estradiol level was determined by using an ELISA kit (**C**). Respective values in bar graphs (mean ± SEM, *n* = 9) not having same alphabetical lowercase (a–e) are different at *p* < 0.05.

Surgically induced estrogen loss for 8 weeks caused a marked atrophy of the uterus (Figure 1A). There was a marked reduction in the uterus in size and wet weight due to OVX (Figure 1B). Administration of 2 mg/kg isoflavone and 618 mg/kg fsCH to OVX mice restored the uterus to its normal size. In addition, fsCH and isoflavone increased the wet weight of OVX mouse uteruses (Figure 1B). It should be noted that 15 mg/kg GPH and 206 mg/kg calcium lactate improved the uterus in size and wet weight (Figure 1A,B). Furthermore, the serum 17β-estradiol level declined in OVX mice, whereas the administration of fsCH and isoflavone significantly enhanced the level (Figure 1C). However, GPH and calcium lactate did not show such effect. Nevertheless, the combination of fsCH and calcium lactate resulted in a synergy effect for the uterus and serum17β-estradiol level (Figure 1B,C).

### 3.2. Effects of fsCH on Bone Minerals and Osteoclast Activation in OVX Mice

There was significant loss of BMD and BMC in mouse femur and tibia tissues observed at 8 weeks after OVX (Table 2). However, the treatment of OVX mice with 206–618 mg/kg fsCH, 2 mg/kg isoflavone, 15 mg/kg GPH, or 206 mg/kg calcium lactate increased femoral and tibial BMD and BMC in mice, as compared to those of the sham-operated control mice (Table 2). When OVX mice were supplemented with 206 mg/kg fsCH and 206 mg/kg calcium lactate at the same time, the increment in BMD and BMC was synergic and the BMD and BMC were nearly restored (Table 2).

**Figure 2:**
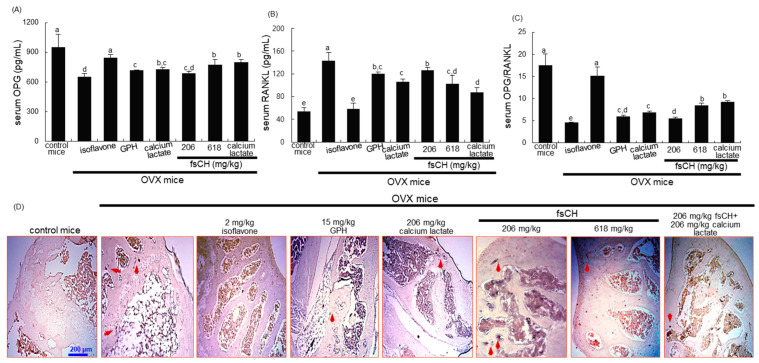
Effects of Pangasius hypophthalmus fish skin hydrolysates (fsCH) on serum osteoprotegerin (OPG)/receptor activator of the nuclear factors κB ligand (RANKL) ratio (**A**–**C**), and TRAP localization in femoral bone tissue sections of OVX mice (**D**). Ovariectomized (OVX) C57BL/6 female mice were orally administrated with 2 mg/kg isoflavone, 15 mg/kg glycine–proline–hydroxyproline tripeptide (GPH), 206 mg/kg calcium lactate, and 206–618 mg/kg Pangasius hypophthalmus fish skin hydrolysates (fsCH) daily for 8 weeks. Serum levels of OPG and RANKL were determined by using ELISA kits (**A**–**C**). Respective values in bar graphs (mean ± SEM, *n* = 9) not sharing an alphabetical lowercase (a–d) are different at *p* < 0.05. The TRAP staining of longitudinal femoral bone tissues was conducted by using a leukocyte acid phosphatase kit (**D**). TRAP-positive osteoclasts (purple) are observed at the femoral trabeculae. Scale bar = 200 μm. Representative images were visualized under light microscopy.

This study examined the serum levels of OPG and RANKL on the eighth week following surgical estrogen deprivation. The serum OPG level was significantly reduced by uterine loss, whereas the serum RANKL level was elevated in OVX mice more than twofold, as compared to that of sham-operated mice (Figure 2A,B). As expected, isoflavone was highly effective in restoring the ratio of serum levels of RANKL and OPG to that shown in normal mice (Figure 2C). When fsCH was administered for 8 weeks after OVX, the serum RANKL level was somehow reduced. In addition, the ratio of serum levels of RANKL and OPG decreased synergically in OVX mice treated with fsCH and calcium lactate at the same time (Figure 2C). Accordingly, fsCH may block osteoclastic activation in osteoporotic bones of OVX mice.

This study attempted to confirm that fsCH inhibited osteoclast formation in bones, as evidenced by TRAP staining. At 8 weeks after OVX, many TRAP-positive osteoclasts (purple-stained, blue arrows) were observed in the femur of OVX mice (Figure 2D). In contrast, the number of dark purple spots decreased in femoral bones of OVX mice treated with isoflavone and fsCH (Figure 2D). Furthermore, the combined treatment of calcium lactate or 206 mg/kg fsCH appeared to lessen osteoclast formation in the femoral bones of OVX mice (Figure 2D). Therefore, fsCH and GPH, as well as isoflavone, may lessen the increase in osteoclastic bone resorption induced by OVX.

### 3.3. Effects of fsCH on Induction of Osteoclastic Markers

**Figure 3:**
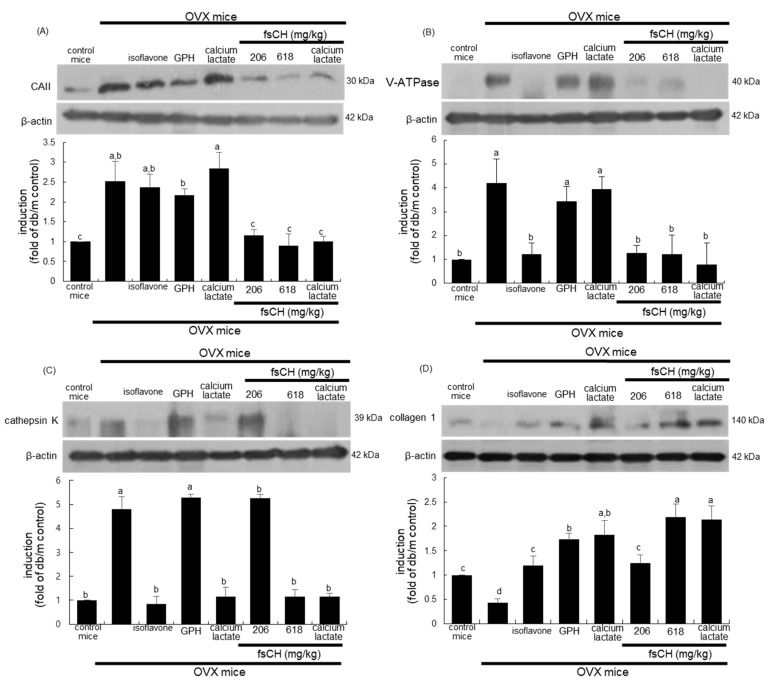
Inhibition of induction of carbonic anhydrase II (CAII, **A**), vacuolar-type H(+)- ATPase (V-ATPase, **B**), and cathepsin K (**C**), and increase in induction of collagen 1 (**D**) by Pangasius hypophthalmus fish skin hydrolysates (fsCH). OVX C57BL/6 female mice were orally administrated with 2 mg/kg isoflavone, 15 mg/kg glycine–proline–hydroxyproline tripeptide (GPH), 206 mg/kg calcium lactate, and 206–618 mg/kg fsCH daily for 8 weeks. Whole bone tissue extracts were subject to SDS-PAGE and Western blot analysis, with a specific antibody against CAII, V-ATPase, cathepsin K, and collagen 1. β-Actin was used as internal control. The bar graphs (mean ± SEM, *n* = 3) represent quantitative results of bands obtained from a densitometer. Values in respective bar graphs not having same alphabetical lowercase (a–d) are different at *p* < 0.05.

This study examined whether fsCH influenced the induction of bone-related biochemical markers in estrogen-deficient mice. Western blot data showed that the estrogen deprivation highly enhanced the induction of CAII and V-ATPase as proton suppliers for bone resorption, and that the induction of cathepsin K is responsible for collagen degradation (Figure 3A–C). The treatment of 206–618 mg/kg fsCH near-completely attenuated the induction of osteoclastic CAII and V-ATPase in OVX mice (Figure 3A,B). Interestingly, GPH and calcium lactate did not show any reduction of CAII and V-ATPase, but the combination of calcium lactate with 206 mg/kg fsCH markedly diminished their induction. Accordingly, fsCH may inhibit bone resorption with acidification of the resorption lacuna. In addition, 618 mg/kg fsCH, similar to 2 mg/kg isoflavone, highly diminished the induction of cathepsin K in OVX mice, inhibiting proteolysis for the removal of the organic matrix (Figure 3C). Herein, the treatment of calcium lactate was effective in lowering cathepsin K induction.

This study further found that the bone levels of collagen type 1 plummeted in OVX mice, as compared to that of the sham-operated mice (Figure 3D). When OVX mice were treated with fsCH and GPH, the bone tissue level of collagen 1 was restored to that of control mice (Figure 3D). The treatment of calcium lactate also increased the collagen 1 level in OVX mice. Accordingly, fsCH may promote the formation of collagenous bone matrix.

### 3.4. Effects of fsCH on Collagen Metabolism by OVX

**Figure 4:**
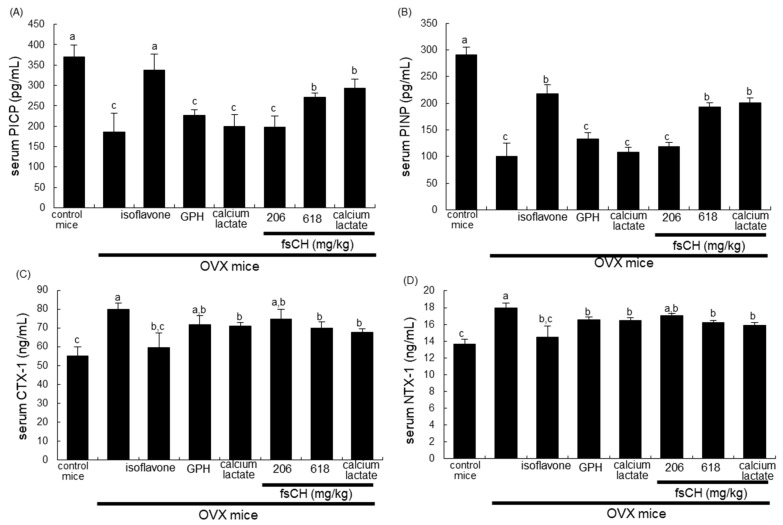
Effects of Pangasius hypophthalmus fish skin hydrolysates (fsCH) on collagen synthesis and degradation in ovariectomized (OVX) mice. OVX C57BL/6 female mice were orally administrated with 2 mg/kg isoflavone, 15 mg/kg glycine–proline–hydroxyproline tripeptide (GPH), 206 mg/kg calcium lactate, and 206–618 mg/kg Pangasius hypophthalmus fish skin hydrolysates (fsCH) daily for 8 weeks. Serum levels of procollagen type 1 carboxy-terminal propeptide (PICP, **A**), procollagen type 1 amino-terminal propeptide (PINP, **B**), carboxy-terminal telopeptide of type 1 collagen (CTX-1, **C**), and amino-terminal telopeptide of type 1 collagen (NTX-1, **D**) were measured by using ELISA kits. Respective values (mean ± SEM, *n* = 3) in bar graphs not sharing an alphabetical lowercase (a, b, c) are different at *p* < 0.05.

The current study investigated beneficial effects of fsCH on collagen formation in OVX mice. The PICP and PINP proteins are liberated in equimolar concentrations into the circulation during the during collagen synthesis [37]. The most abundant protein in bone is type 1 collagen. The OVX reduced serum levels of PICP and PINP in mice, as evidenced by ELISA (Figure 3A,B). The treatment with 618 mg/kg fsCH and isoflavone significantly elevated the serum levels of these propeptides reduced by OVX. In addition, orally supplying combined fsCH and calcium lactate to mice synergically increased serum levels of PICP and PINP (Figure 4A,B). Accordingly, the presence of fsCH may ameliorate collagen synthesis in bone deteriorated by estrogen deprivation.

This study further examined whether fsCH influenced serum levels of CTX-1 and NTX-1, both released during collagen degradation. The release of CTX-1 and NTX-1 into the circulation was highly enhanced in OVX mice (Figure 4C,D). The increased release of both biomarkers responsible for the collagen breakdown was reduced by the treatments with fsCH, isoflavone, and calcium lactate (Figure 4C,D). Thus, fsCH was effective in boosting collagen-mediated osteogenesis in OVX-induced osteoporotic bone.

### 3.5. Formation of Trabecular Bone by fsCH in OVX Mice

**Figure 5:**
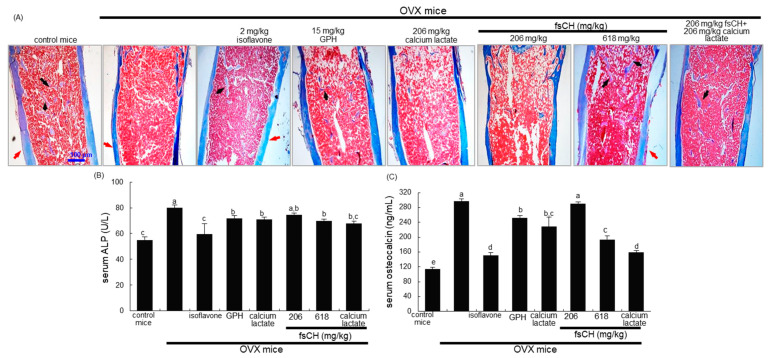
Inhibition of trabecular bone loss by Pangasius hypophthalmus fish skin hydrolysates (fsCH), and alterations in serum levels of alkaline phosphatase (ALP) and osteocalcin. OVX C57BL/6 female mice were orally administrated with 2 mg/kg isoflavone, 15 mg/kg glycine–proline–hydroxyproline tripeptide (GPH), 206 mg/kg calcium lactate, and 206–618 mg/kg fsCH daily for 8 weeks. Decalcified femoral bones of ovariectomized (OVX) mice were H&E-stained (**A**). Pink-stained mineralized bone, cortical bone outside, columns of trabecular bone inside. Scale bar = 100 μm. The serum level of ALP was measured by incubating with *p*-nitrophenyl phosphate and MgCl_2_ in Tris-HCl buffer. The absorbance was read at λ = 405 nm (**B**). The serum level of osteocalcin was measured by using an ELISA kit (**C**). Respective values (mean ± SEM, *n* = 3) in bar graphs not sharing an alphabetical lowercase (a-e) are different at *p* < 0.05.

In the eighth week after ovariectomy, histological sagittal sections of the femur showed that the bony meshwork was observed in the metaphysis and diaphysis of OVX mice, as compared with the sham-operated control mice (Figure 5A). In contrast, the trabecular bones were newly formed in the metaphysis and diaphysis of OVX mice treated with isoflavone and fsCH (black arrows, Figure 5A). Consistent with the histological findings and TRAP staining data (Figure 2D), there was a significant increase in BMD in the femur bones of 618 mg/kg fsCH- or isoflavone-treated mice compared with that of OVX-alone mice (Table 2). On the other hand, the treatment of GPH, calcium lactate or 206 mg/kg fsCH did not exhibit such favorable outcomes in the femoral bones of OVX mice (Figure 5A). However, oral administration of the combined fsCH and calcium lactate to mice appeared to enhance the formation of trabecular bones (Figure 5A). Furthermore, thin cortical bones (red arrows) were observed in the femur of OVX mice (Figure 5A). In marked contrast, the cortical bones of OVX mice treated with 618 mg/kg fsCH or isoflavone were indistinguishable from those of the sham control animals. Accordingly, the presence of fsCH may ameliorate the new bone formation failed by estrogen deficiency.

### 3.6. Inhibition of Secretion of ALP and Osteocalcin into Circulation by fsCH

The current study investigated the serum ALP levels of OVX mice. The ALP secretion increased significantly in OVX mice, compared to the sham-operated mice (Figure 5B). In OVX mice receiving 2 mg/kg isoflavone, the serum level was reversed. Similarly, 618 mg/kg fsCH decreased the ALP secretion into the circulation (Figure 5B). Furthermore, the combination of calcium lactate and fsCH displayed marked synergic effects on preservation of bone ALP, reflecting increased osteoblastic activity in bones.

This study further examined osteocalcin levels in the serum of OVX mice. The serum osteocalcin level was significantly enhanced due to estrogen deficiency (Figure 5C). In OVX mice treated with 2 mg/kg isoflavone or 618 mg/kg fsCH, the serum level highly decreased. Although 206 mg/kg fsCH alone did not reduce the serum level significantly, its combination with 206 mg/kg calcium lactate highly attenuated the increase in serum osteocalcin level (Figure 5C). Accordingly, fsCH may enhance bone formation and mineralization triggered by non-collagenous osteocalcin in bones.

### 3.7. Effects of fsCH on Osteoblastogenesis

**Figure 6:**
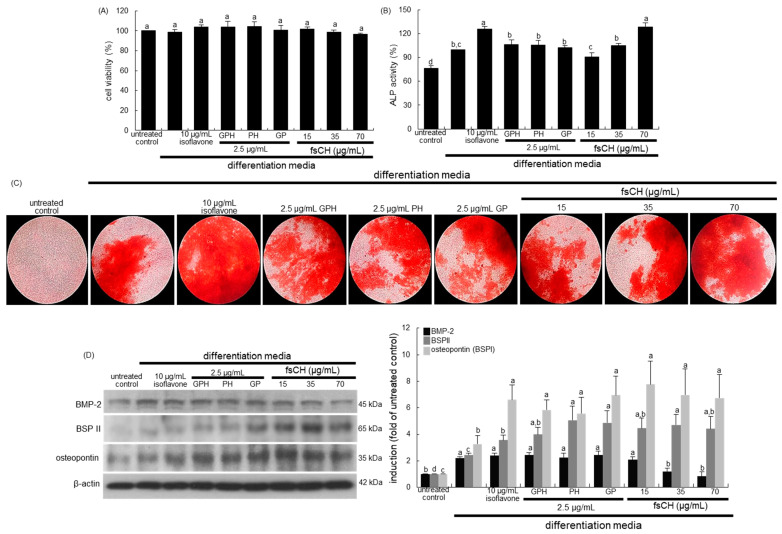
MC3T3-E1 cell toxicity of Pangasius hypophthalmus fish skin hydrolysates (fsCH, **A**), upregulation of alkaline phosphatase (ALP) activity (**B**), calcium nodule formation by fsCH (**C**), and induction of non-collagenous matrix proteins (**D**). MC3T3-E1 cells were cultured for up to 21 days with 10 μg/mL isoflavone, 2.5 μg/mL glycine–proline–hydroxyproline tripeptide (GPH), 2.5 μg/mL proline–hydroxyproline dipeptide (PH), 2.5 μg/mL glycine–proline dipeptide (GP), and 15–70 μg/mL fsCH in differentiation media. Cell viability was measured by MTT assay (**A**). Bar graphs for viability (mean ± SEM, *n* = 3) was expressed as percentage of cell survival compared to untreated cells. MC3T3-E1 cells were cultured in differentiation media in the absence or presence of isoflavone, GPH, PH, GP, and fsCH for seven days. The ALP activity (**B**, mean ± SEM, *n* = 6) was measured at λ = 405 nm. Matrix mineralization was measured by Alizarin red S staining (**C**). Microphotographs were representative of 21 day-grown osteoblasts on the wells. Heavy reddish staining of Alizarin red S is proportional to the area of mineralized matrix in osteoblastic MC3T3-E1 cells. The calcium nodules were visualized under light microscopy (5 separate experiments). Magnification: 40-fold. Whole cell lysates were subject to SDS-PAGE and Western blot analysis with a specific antibody against bone morphogenetic protein-2 (BMP-2), bone sialoprotein II (BSPII), and osteopontin (**D**). β-Actin was used as internal control. The bar graphs (mean ± SEM, *n* = 3) represent quantitative results of bands obtained from a densitometer. Values in respective bar graphs not having same alphabetical lowercase (a–d) are different at *p* < 0.05.

This study investigated whether fsCH and fish skin oligopeptides stimulated the osteogenesis of cultured osteoblasts. There was no significant toxicity of fsCH and oligopeptides, including isoflavone, observed in MC3T3-E1 cells incubated in differentiation media for three days (Figure 6A). ALP is expressed on the osteoblast surface during differentiation as an early marker gene, and facilitates bone matrix maturation and mineralization [38]. When MC3T3-E1 cells were cultured for seven days in differentiation media containing 50 μg/mL ascorbic acid, and 100 nM dexamethasone, the ALP activation was highly elevated (Figure 6B). Such elevation was further enhanced in differentiation media with 10 μg/mL isoflavone and 70 μg/mL fsCH. The oligopeptides of GPH, PH, and GP tended to increase the activation (Figure 6B).

The formation of calcium nodules is one of the characteristics of mature osteoblasts [39]. There was no noticeable calcium deposit in undifferentiated MC3T3-E1 cells, while strong reddish staining was observed in MC3T3-E1 cells differentiated for 21 days, evidenced by Alizarin red S staining (Figure 6C). In differentiated osteoblasts treated with fsCH, the calcium deposition was enhanced in a dose-dependent manner. It should be noted that isoflavone was a potent agent promoting osteoblastic differentiation and matrix mineralization (Figure 6C).

This study investigated whether fsCH ameliorated the expression of osteogenic proteins of BMP-2, BSPII, and osteopontin during 21 day-osteoblastic differentiation. The cellular BMP-2 expression was enhanced significantly at the early–mid stage of differentiation [40]. The treatment of pre-osteoblastic cells with isoflavone, GPH, PH, and GP in osteogenic media did not influence the induction of BMP-2 (Figure 6D). When cells were treated with 15–70 μg/mL fsCH, the BMP-2 induction was diminished. The non-collagenous BSPII and osteopontin, also known as BSPI, are necessary for the initiation of bone mineralization in the bone matrix [41,42]. Our previous study showed that the cellular expression of BSPII and osteopontin highly increased at the mid-late stage of differentiation [43]. The expression of BSP II and osteopontin was enhanced at 12–15 days after osteogenic differentiation, and further such induction of these non-collagenous proteins was highly promoted by treating all of the collagen peptides and fsCH, as well as isoflavone, to 15-day-differentiating MC3T3-E1 cells (Figure 6D). Accordingly, both non-collagenous proteins of BSP II and osteopontin were induced by fsCH ahead of the mineralization front as mineral crystal nucleators.

## 4. Discussion

**Figure 7:**
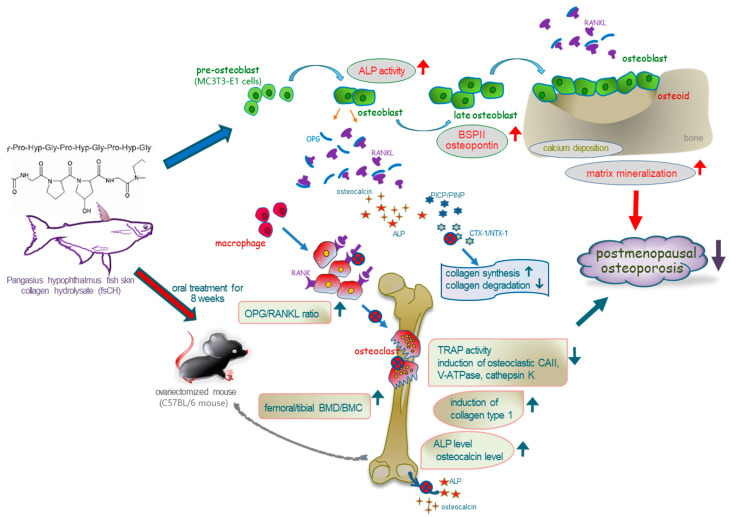
Schematic diagram showing effects of Pangasius hypophthalmus fish skin hydrolysates (fsCH) on bone loss and osteoblastogenesis in ovariectomized mouse model. The arrows indicate activation by fsCH. ALP, alkaline phosphatase; BMD, bone mineral density; BSPII, bone sialoprotein II; CAII, carbonic anhydrase II; CTX-1, carboxy-terminal telopeptide of type 1 collagen; GPH, glycine–proline–hydroxyproline; NTX-1; amino-terminal telopeptide of type 1 collagen; OPG, osteoprotegerin; PICP, procollagen type 1 carboxy-terminal propeptide; PINP, procollagen type 1 amino-terminal propeptide; TRAP, tartrate-resistant acid phosphatase; RANK, receptor activator of nuclear factor-κB; RANKL, RANK ligand; V-ATPase, vacuolar-type H(+)- ATPase. The symbol → indicates activation manifested by fsCH and the symbol ⊗ indicates sites of inhibition.

The nine major findings were extracted from this study employing fsCH. (1) Surgical estrogen loss of mice for 8 weeks caused uterus atrophy and reduced serum17β-estradiol levels, which was highly ameliorated by the administration of 618 mg/kg fsCH and 2 mg/kg isoflavone to mice. (2) The ratio of serum levels of OPG and RANKL was synergically elevated when fsCH and calcium lactate were simultaneously administered to OVX mice. (3) The treatment of OVX mice with fsCH, isoflavone, GPH, or calcium lactate increased the reduced BMD and BMC in femur and tibia tissues, along with the disappearance of TRAP-positive osteoclasts. (4) Supplementing fsCH to OVX mice reduced osteoclastic induction of CAII, V-ATPase, and cathepsin K in OVX bones, and elevated bone levels of collagen 1. (5) The treatment with fsCH and isoflavone enhanced the serum levels of PICP and PINP reduced by OVX, whereas the release of CTX-1 and NTX-1 into the circulation was reduced in OVX mice treated with fsCH, isoflavone, and calcium lactate. (6) The trabecular bones were newly formed in the metaphysis and diaphysis of OVX mice treated with isoflavone and fsCH, but not with calcium lactate. (7) Similar to isoflavone, fsCH enhanced the bone levels of ALP and osteocalcin in OVX mice by reducing their secretion into the circulation. (8) Similar to 10 μg/mL isoflavone, 70 μg/mL fsCH enhanced the ALP activity and calcium deposition in osteoblastic MC3T3-E1 cells. (9) The treatment of fsCH enhanced the induction of non-collagenous matrix proteins of BSPII and osteopontin after 15-day-differentiation. Accordingly, the presence of fsCH may restore bone formation and collagen synthesis failed by surgical estrogen deficiency through enhancing osteoblastic activation and matrix mineralization (Figure 7).

Osteoporosis is a metabolic bone disorder characterized by a microarchitectural deterioration of bone tissues that leads to increased bone fragility and fractures, especially in the proximal femur, vertebrae, and distal radius [11,12]. The imbalance between bone formation and resorption has detrimental effects on trabecular bone and cortical bone, resulting in trabecular thinning and loss of connectivity, and cortical thinning and porosity, respectively [44]. Estrogen deficiency resulting in postmenopausal osteoporosis, the most common type of osteoporosis, causes an increase in bone remodeling involving all types of bone cells [4,11]. Osteoporotic fractures in postmenopausal women can be costly, and result in disability or death. This study found that surgically induced estrogen loss for 8 weeks caused marked uterus atrophy and a significant reduction in BMD and BMC in mouse femur and tibia tissues, producing trabecular and cortical thinning and porosity. A total of 8 weeks after ovariectomy, the bony meshwork appeared in the femoral metaphysis and diaphysis of the OVX mice. This study showed that surgical estrogen deprivation led to both RANKL upregulation and OPG downregulation in serum levels by possibly accelerating osteoclastogenic bone loss. In addition, OVA resulted in increased serum levels of ALP and osteocalcin, as well as decreased bone levels of these osteoblastic markers. The interplay of the RANK/RANKL/OPG system in osteoclasts and osteoblasts and the role of ALP and osteocalcin may act as mechanism(s) involved in OVX-induced bone remodeling. Improved knowledge of the cellular basis for postmenopausal osteoporosis helps to develop new drugs targeting key mechanistic pathways [45].

Preventive strategies to improve bone health include adequate calcium, vitamin D, weight-bearing exercise, and abstaining from smoking [8,46]. Increased intake of dietary carotenoids, including β-carotene and β-cryptoxanthin, is effective in lowering osteoporotic fracture risk [9,10]. Potential pharmacological drugs for postmenopausal osteoporosis are therapies that improve bone strength, leading to efficient recovery of bone mass [17,18]. Pharmacological therapies are used in patients with osteoporosis in order to reduce the risk of fracture and increase BMD [47,48]. Currently used drugs for pharmacological therapies, such as bisphosphonates, denosumab, and teriparatide, are licensed to reduce fracture risk by slowing down bone resorption and/or by stimulating bone formation [4,6,7]. However, the effects of denosumab are not sustained when treatment is discontinued [46]. The long-term use of these current drugs is limited by various adverse effects [47,49]. On the other hand, hormone replacement therapy is an effective treatment for the prevention and treatment of adverse postmenopausal alterations [13,14]. However, estrogen replacement therapy increases the risk of breast cancer and malignancy; as such, its wide usage is not recommended [15,16]. To reduce such adverse effects, individual variability, concentration, and timing of the administration of drugs, as well as the mechanisms of action of the drug, should be carefully considered. Therefore, there is a need to develop novel drugs that effectively inhibit osteoporosis while minimizing adverse effects.

The increased risk of long-term hormone therapy in women with postmenopausal osteoporosis requires the use of harmless and affordable alternative therapies [19]. The use of complementary therapies is fairly widespread among women in order to alleviate postmenopausal osteoporosis [49]. Potential agents inhibiting osteoclastic activation may be effective in combating estrogen loss-induced resorptive bone diseases. The treatment of β-cryptoxanthin inhibits osteoclastic activation and the reduction of bone volume in OVX mice [9]. The polyphenols of phloretin and EGCG inhibit estrogen deficiency-induced osteoclastogenic bone loss in mice [24,25,26]. Nonsteroidal isoflavones act as phytoestrogens in mammals, and have estrogen-like biological activity primarily through binding to estrogen receptors [20,21,22]. This study showed that oral administration of isoflavone inhibited uterus atrophy with an increase in serum 17β-estradiol levels, and increased the BMD and BMC in femur and tibia tissues of OVX mice. In addition, isoflavone enhanced the ratio of serum OPG/RANKL in OVX mice, possibly improving OVX-induced bone remodeling. Similar to isoflavone, fsCH containing collagen peptides of GPH, GP, and PH was effective in counteracting estrogen loss-induced uterus atrophy and osteoclastic bone loss. This study revealed that fsCH enhanced the formation of trabecular bones and collagenous matrix in the metaphysis and diaphysis of OVX mice through reducing expression of the osteoclastic biomarkers of CAII, V-ATPase, and cathepsin K. Other investigations have shown that collagen hydrolysates have a beneficial effect on osteoarthritis and osteoporosis [31,32]. However, the action mechanisms of fsCH on bone health in the postmenopausal state have not yet been defined.

This study conducted inter-comparative experiments for anti-osteoporotic activity among fsCH, isoflavone, GPH tripeptide, and calcium lactate. The GPH treatment was effective in eliminating estrogen loss-elicited detrimental outcomes in bone. GPH, hydrolyzed to PH and GP, appeared to increase the trabecular bone formation within the metaphysis and diaphysis of OVX mice through blocking collagen degradation, rather than increasing its synthesis. In addition, GPH was not effective in inhibiting osteoclastic bone resorption, but it appeared to stimulate osteoblastic differentiation and matrix mineralization by non-collagenous osteogenic proteins. Collagen comprises polyproline-like helices with a repeating sequence of GPH that can hydrolyze to dipeptides such as PH by intestinal epithelium-bound aminopeptidase [35,36,50]. The fsCH treatment stimulated osteoblastogenesis of mature osteoblasts in a dose-dependent fashion, which might contribute to the inhibition of OVX-triggered bone loss. Further, the GPH-hydrolyzed dipeptides of GP and PH, as well as GPH, tended to increase osteoblastogenic calcium deposition. In addition to single therapies with drugs, combination therapy and sequential therapy have been under investigation to treat osteoporosis more effectively [51,52]. Interestingly, low-dose combination of fsCH and calcium lactate exhibited a pharmacological synergy effect on the anti-osteoporotic activity of calcium lactate, especially through inhibiting the induction of bone loss-associated osteoclastic proteins, which may be useful for treating postmenopausal osteoporosis. Nevertheless, the evidence for the combination therapy of fsCH and calcium lactate targeting bone formation is still limited in this pre-clinical study performed with BMD as the endpoint.

In summary, the current report demonstrated that fsCH abrogated estrogen deficiency-induced bone loss. The oral administration of fsCH improved uterus atrophy induced by OVX, and simultaneously restored the low BMD of femur and tibia tissues. Additionally, the constituent tripeptide GPH of fsCH enhanced the BMD in OVX-exposed femur and tibia tissues with the increase in trabecular bone formation through enhancing osteoblastogenic activation. Decreased osteoclastic activation and increased osteoblastogenic differentiation and mineralization contributed to the inhibition of bone loss by fsCH containing GPH and PH in postmenopausal osteoporosis. It should be noted that the lack of osteogenic activity of calcium lactate in postmenopausal osteoporosis was complemented by fsCH in a synergetic manner. Unfortunately, this study did not examine the formation of hydroxyapatite crystals and matrix vesicle-mediated bone mineralization with fsCH. Finally, similar to isoflavone, fsCH may be a natural compound targeting against postmenopausal osteoporosis and pathological osteoresorptive disorders. However, the action mechanisms of fsCH on bone health need to be defined on a clinical level.

## Figures and Tables

**Table 1:** Effects of various substances on plasma cholesterol and triglyceride in ovariectomized mice.

	Animals	Control Mice	OVX Mice		OVX Mice				
				2 mg/kg Isoflavone	15 mg/kg GPH	206 mg/kg Calcium Lactate	fsCH		
Parameters							206 mg/kg	618 mg/kg	206 mg/kg Calcium Lactate
total cholesterol		79.8 ± 5.5 ^b^	91.2 ± 2.5 ^a^	84.2 ± 3.6 ^a,b^	94.6 ± 3.17 ^a^	89.4 ± 3.9 ^a^	90.6 ± 3.0 ^a^	95.6 ± 2.8 ^a^	93.4 ± 3.5 ^a^
triglyceride		35 ± 4.3 ^c^	51.4 ± 9.6 ^a^	43 ± 2.2 ^b^	53.6 ± 6.0 ^a^	34 ± 2.6 ^c^	34.4 ± 2.7 ^c^	51.6 ± 3.0 ^a^	42.6 ± 7.1 ^a,b^
HDL-C		47.2 ± 7.1 ^a,b^	44.6 ± 2.5 ^b^	50 ± 3.9 ^a^	53.8 ± 3.4 ^a^	48.4 ± 2.9 ^a,b^	48.2 ± 3.1 ^a,b^	51 ± 1.6 ^a^	51 ± 1.4 ^a^
LDL-C		25.6 ± 5.4 ^b^	36.3 ± 6.1 ^a^	25.6 ± 2.7 ^b^	30.08 ± 1.0 ^a,b^	34.2 ± 0.8 ^a^	35.5 ± 3.4 ^a^	34.3 ± 2.6 ^a^	33.9 ± 2.5 ^a^

C57BL/6 female mice (11 weeks of age, 20–25 g) were surgically ovariectomized (OVX) and orally administrated with 2 mg/kg isoflavone, 15 mg/kg glycine–proline–hydroxyproline tripeptide (GPH), 206 mg/kg calcium lactate, and 206–618 mg/kg Pangasius hypophthalmus fish skin hydrolysates (fsCH) daily for 8 weeks. Respective values in same row (mean ± SEM, *n* = 9) not sharing an alphabetical lowercase (a, b, c) are different at *p* < 0.05.

**Table 2:** Osteogenic activity of various substances in ovariectomized mice.

	Animals	Control Mice	OVX Mice	OVX Mice					
				2 mg/kg Isoflavone	15 mg/kg GPH	206 mg/kg Calcium Lactate	fsCH		
Parameters							206 mg/kg	618 mg/kg	206 mg/kg Calcium Lactate
femur	BMD	89.3 ± 0.5 ^a^	72.6 ± 0.4 ^d^	84.6 ± 0.5 ^b^	81.6 ± 0.3 ^c^	79.6 ± 0.3 ^c^	78.5 ± 0.2 ^c^	82.5 ± 0.5 ^b,c^	84.4 ± 0.4 ^b^
	BMC	18.8 ± 0.3 ^a^	14.9 ± 0.2 ^c^	17.3 ± 0.9 ^a,b^	16.5 ± 0.4 ^b^	16.1 ± 0.3 ^b^	16.4 ± 0.4 ^b^	17.2 ± 0.4 ^a,b^	17.1 ± 0.4 ^a,b^
	area	20.8 ± 0.3	20.9 ± 0.3	20 ± 1.2	20.5 ± 0.4	20.6 ± 0.4	21 ± 0.5	20.8 ± 0.4	19.9 ± 0.6
tibia	BMD	63.8 ± 0.3 ^a^	51.2 ± 0.3 ^d^	62.2 ± 0.1 ^a^	57.8 ± 0.2 ^b^	56.8 ± 0.4 ^b^	54.6 ± 0.4 ^c^	59.9 ± 0.3 ^a,b^	61.4 ± 0.3 ^a^
	BMC	9.4 ± 0.2 ^a^	7.4 ± 0.2 ^d^	8.7 ± 0.3 ^b^	8.1 ± 0.2 ^c^	8.2 ± 0.1 ^c^	8 ± 0.0 ^c^	8.8 ± 0.1 ^b^	9 ± 0.2 ^b^
	area	14.8 ± 0.2	14.4 ± 0.2	14 ± 0.6	14.3 ± 0.3	14.8 ± 1.3	14.3 ± 0.2	14.4 ± 0.2	14.4 ± 0.2

Ovariectomized (OVX) C57BL/6 female mice were orally administrated with 2 mg/kg isoflavone, 15 mg/kg glycine–proline–hydroxyproline tripeptide (GPH), 206 mg/kg calcium lactate, and 206–618 mg/kg Pangasius hypophthalmus fish skin hydrolysates (fsCH) daily for 8 weeks. The bone mineral density (BMD, mg/cm^2^), bone mineral content (BMC, mg), and bone area (cm^2^) of mouse tissues of femurs and tibiae were determined by using a PIXImus mouse densitometer. Respective values in same row (mean ± SEM, *n* = 9) not sharing an alphabetical lowercase (a-d)are different at *p* < 0.05.

## Data Availability

All the data presented in this study are included in the article.

## Abbreviations

ALP	alkaline phosphatase
BMD	bone mineral density
BMP-2	bone morphogenetic protein 2
BSPII	bone sialoprotein II
CAII	carbonic anhydrase II
CTX-1	carboxy-terminal telopeptide of type 1 collagen
fsCH	Pangasius hypophthalmus fish skin collagen hydrolysate
GP	glycine–proline
GPH	glycine–proline–hydroxyproline
NTX-1	amino-terminal telopeptide of type 1 collagen
OPG	osteoprotegerin
OVX	ovariectomy
PICP	procollagen type 1 carboxy-terminal propeptide
PH	proline–hydroxyproline
PINP	procollagen type 1 amino-terminal propeptide
RANKL	receptor activator of nuclear factor *kappa*-Β ligand
TRAP	tartrate-resistant acid phosphatase
V-ATPase	vacuolar-type H(+)- ATPase
